# Chromosomal microarray analysis, or comparative genomic hybridization: A high throughput approach

**DOI:** 10.1016/j.mex.2015.11.005

**Published:** 2015-12-02

**Authors:** Mohammad Haeri, Violet Gelowani, Arthur L. Beaudet

**Affiliations:** a Department of Molecular & Human Genetics, One Baylor Plaza, Houston, TX, United States; b Department of Pathology & Immunology, Baylor College of Medicine, One Baylor Plaza, Houston, TX, United States

**Keywords:** Copy number variants, Comparative genomic hybridization, Chromosomal microarray analysis

## Abstract

Pathological copy number variants (CNVs) and point mutations are major genetic causes of hundreds of disorders. Comparative genomic hybridization (CGH) also known as chromosomal microarray analysis (CMA) is the best available tool to detect copy number variations in chromosomal make up. We have optimized several different protocols and introduce a high-throughput approach to perform a cost-effective, fast, high-throughput and high-quality CMA. We managed to reach to high quality arrays with 17 ± 0.04 (mean ± SD, *n* = 90) Derivative Log Ratio (DLR) spread, a measure of array quality (<0.20 considered as excellent) for our arrays. High-throughput and high-quality arrays are gaining more attention and the current manuscript is a step forward to this increasing demand.

• This manuscript introduces a low cost, fast, efficient, high throughput and high-quality aCGH protocol;
• This protocol provides specific instructions and crucial detail for processing up to 24 slides which is equal to 48, 96, or 192 arrays by only one person in one day;
• This manuscript is accompanied with a step-by-step video.

## Method details

Chromosomal microarray analysis (CMA) also known as comparative genomic hybridization (CGH) and SNP arrays are routine clinical tools for the evaluation of patients with various diseases and congenital malformations. Surprisingly, the extent of variation in copy number in the human genome outnumbers the heterogeneity in sequence variation. There are several databases providing data for CNVs in healthy controls and symptomatic individuals such as, Database of Genomic Variants, (DGV); International Collaboration for Clinical Genomics, (ICCG); Decipher; and dbVar, a database of genomic structural variation. Many human disorders could be caused by CNVs such as, cognitive impairments, behavioral abnormalities including autism, schizophrenia, bipolar disorder, epilepsy and congenital malformations in heart and other organs [1], [2], [3], [4], [5], [6], [7], [8].

A critical step in producing a reliable, low-noise image/data with a high signal to noise ratio is to perform a high quality aCGH which is well known to be time-consuming. We introduce a fast and high-throughput approach to process numerous arrays without compromising the quality of the array. This approach provides a fast, efficient, high-throughput and low cost aCGH for commercially available or custom designed arrays. In this protocol we used the custom designed Baylor College of Medicine (BCM) arrays available to public by Agilent Technology (Santa Clara, CA).

Baylor College of Medicine arrays are whole-genome exon-targeted oligonucleotide microarrays available through Agilent in the following versions: V8 (180 K), V9 (400 K), and V10 (400 K) OLIGO with 60 K SNP probes enabling detection of single exon copy-number variants and regions of absence of heterozygosity (AOH) [9], [10], (http://www.bcm.edu/geneticlabs/) [11]. Larger arrays cover more genes associated with covered disorders. The latest versions cover the majority of defined inherited disorders including developmental or intellectual disorders (DD/ID), autism spectrum disorders (ASDs), seizures, dysmorphic features, heart defects, speech delay, attention deficit hyperactivity disorder (ADHD) among others. Several probes are used to interrogate the arrangement of a single gene [9], [10], [11], [12], [13].

The purpose of this manuscript is to give important details of the process and to introduce a low-cost, fast, efficient, high-throughput and high-quality aCGH protocol. These qualities rarely occur simultaneously. More specifically: (a) this protocol is at least 60% less costly compared to available aCGH DNA preparation kits in the market. (b) It is high-throughput as one can process 24 slides, which is equal to 48 × 400 K arrays or 196 × 60 K arrays in a single run with regular lab instruments and just one person. The bottleneck for processing more slides in a run is the capacity of the common available rotating oven which is 24 slides. Obviously, with a higher capacity oven or multiple ovens more arrays can be processed. (c) It is fast and efficient because one can start from the first step and finish the hybridization on the same day within regular lab hours. (d) It is high quality as we managed to attain excellent quality for the majority of our arrays.

The key feature of the current protocol for a fast, cost effective, and efficient result is the optimization of previous methods to use thermocycler with 96-well plate or string PCR tubes and also multi-channel pipettors. Additionally, we carefully described minor elements that can significantly increase the quality of signals when applied together. Although our goal is a high-throughput protocol for aCGH, SNP, and combined SNP + aCGH arrays, this protocol can be efficiently used for as low as 4 slides while it is still cost-effective. Our intention is to demonstrate simple and complicated steps using common lab instruments. Obviously, use of 96-chanel pipettors or automated robotic liquid dispensers will make the process even faster. However, this equipment is not readily accessible for small- and medium-sized laboratories. Finally, every protocol has its limitation. This protocol is designed for processing 4–24 slides (can be equal to 48, 96, or 192 arrays when using 24 slides) by one person, and can be performed in one day (5–8 h) based on the experience and equipment used by the operator. We commonly used Agilent arrays in this protocol; however, the same protocol can be used for other platforms with minor adjustment. Accordingly, we used the same protocol for custom-made arrays and achieved excellent results. We have successfully utilized the same protocol for dual aCGH/SNP array made by Agilent. An accompanying video with this protocol simplifies performing sensitive key steps of the procedure. In order to facilitate the use of this protocol for small laboratories, simple and common lab equipment is utilized. Some steps were intentionally performed slower than real in the video to make it more comprehensible. Other parts of the video are fast forwarded for the sake of time.

In order to speed up the process and make it high-throughput, we first identified every rate limiting step along the process. In regular protocols, the use of 1.5 ml tubes with heat blocks, water-bath and 1.5 ml purification units make the process very slow and error prone. We set the stage for using similar tube formats at each step to maximize the speed and efficiency and to reduce possible errors along the way. This was achieved by using 96-well plates PCR, multichannel pipettors and 96-well plate purification units. Using a 96-well pipettor (INTEGRA Biosciences AG) will remarkably increase the speed of the process.

We used both strip PCR tubes and 96-well plate PCR tubes. The recommended 96-well plate is the PCR 96-well plate raised rim (Simport Amplate™ Raised Rim Thin Wall PCR Plates, part # 30-T323101Y or 96-well Tall-Chimney PCR plate, Fisherbrand, part #: 14-230-242) for which a strip cap is recommended (Strip caps, Neptune, CA 92121, part # 3727.12S.X) to prevent fluid loss at boiling temperature.

For a lab interested in performing 24 slides at once, here is our recommendation.

1- Use a 96-channel pipettor to distribute DNA into 96-well plate with raised rim.
2- Use a 96-well plate with raised rim for initial enzyme/heat denaturation step.
3- Use a 96-channel pipettor to transfer the labeled DNA to 96-well plate purification unit.
4- Use a 96-channel pipettor to elute the purified labeled DNA form the 96-well plate purification unit.
5- Use a 96-well plate (340 μl Raised Rim Thin Wall PCR Plates) for denaturation followed by 37 °C incubation.
6- Use thermocycler for denaturation followed by 37 °C incubation.

*Note*: For a high-throughput approach we recommend using a thermocycler in all steps, however, when using 340 μl PCR tubes or 96-well plates, one should make sure that the thermocycler accepts the large volume tubes/plate.

## Materials and methods

Please note that this manuscript is accompanied with a detailed step-by-step video. Please follow the link to view our video: This video is useful to demonstrate critical steps in performing the experiment that is difficult to describe in text.

In order to start a CMA, one microgram genomic DNA of each patient and control subject is required. The genomic DNA can be fragmented either by restriction enzyme digestion or by heat fragmentation. In enzyme digestion the genomic DNA will be digested by restriction enzymes. Alternatively, heat fragmentation can be utilized before the labeling step.

Heat fragmentation is carried out in less than 15 min and can be combined with the addition of random primers to make the procedure faster without affecting the fragmentation step.

Use heat-fragmentation or enzyme digestion of DNA for aCGH array slides. Heat-fragmentation is easier and faster. Please note that if you use a SNP array or SNP + aCGH array you must use the enzyme digestion protocol. The heat-fragmentation and enzyme digestion protocols described below are for 8 × 60 K Agilent arrays.

### Enzyme digestion

1.1 Add 1.0 μg of genomic DNA to each PCR strip tube and bring the total volume to 12 μl with PCR grade water.
1.2 Add the following reagents to each PCR tube: 0.25 μl PCR grade water, 0.25 μl BSA, 0.5 μl AluI, 0.5 μl RsaI, and 1.5 μl buffer. You can prepare a master mix for the water, BSA, AluI, RsaI, and buffer and dispense 3 μl of mastermix to each tube from step 1.1. After this step, the total reaction volume will be 15 μl.
1.3 Set up the following program in your thermocycler, place the tubes into the thermocycler, and run the program: 37 °C for 2 h and 65 °C for 20 min, then 4 °C.
1.5 Confirm that the genomic DNA was properly digested by running 2 μl of the digested product on a 1.2% agarose gel stained with ethidium bromide. A smear should be visible mostly around 200–500 bps.
1.6 Store the digested genomic DNA at −20 °C or proceed to step 1.7.
1.7 (If samples were previously frozen thaw the samples at room temperature and then place them on ice.) Spin down the PCR tubes for 5 s.
1.8 Transfer 10.5 μl of the digested DNA to a new PCR tube.
1.9 Add 10.5 μl of 2.5× Random Primers to each PCR tube containing the 10.5 μl of digested genomic DNA (see step 1.8). The total reaction volume will be 21 μl after this step.
1.10 Mix by flicking. Spin down the tubes for 5 s and put the tubes in the thermocycler at 95 °C for 5 min. While the program is running prepare a bucket with ice water. A few seconds before the program finishes, abort the program, remove the tubes from the thermocycler and place the tubes immediately on ice-water for at least 5 min. Proceed with Fluorescent labeling of genomic DNA.

Alternatively, the DNA can be fragmented by heat-fragmentation as the following steps:

### Heat fragmentations of the genomic DNA

2.1 Add 1.0 μg of genomic DNA to each PCR tube and bring the total volume to 21 μl with PCR grade water.
2.2 Add 20 μl of 2.5× random primers to each PCR tube and close the cap.
2.3 Mix by flicking and spin down for 5 s.
2.4 Set up the following program in your thermocycler, place the tubes into the thermocycler, and run the program. While the program is running prepare a bucket with ice water.

**Table:** 

100 °C	3 min	Denaturation
0 °C	5 min	
100 °C	5 min	Denaturation (2nd)

The lid temperature is 105 °C.

2.5 A few seconds before the last run is finished remove the PCR tubes from the thermocycler and place the tubes immediately on ice, soaked in water, and keep it on the ice for 5 min while it is covered by aluminum foil.
2.6 Proceed with Fluorescent labeling of genomic DNA.

### Fluorescent labeling of genomic DNA

3.1 Spin down the tubes for 5 s and place them back on ice.
3.2 Prepare a master mix for each dye. Use Cy5 (stained blue by the manufacturer, with excitation peak: ∼650 nm, and emission peak: ∼670 nm) for the test DNA and Cy3 (stained red by the manufacturer, with excitation peak: ∼550 nm, and emission peak: ∼570 nm) for the control DNA as it is described in Table 1. Scale up for more reactions.
3.3 Follow the order while adding the components and keep the PCR tubes on ice until the next step.
3.4 If you used heat fragmentation add 9.0 μl of the Labeling Master Mix to each PCR tube. If you used our enzyme digestion protocol use only 4.5 μl of the Labeling Master Mix. Mix by flicking. Spin down the PCR tubes for 5 s.
3.5 Place the PCR tubes in the thermocycler and incubate for 2 h at 37 °C and set the lid temperature at 39 °C.
3.6 Right after the 2-h incubation at 37 °C, keep the tubes in the thermocycler and set the temperature at 65 °C and the cap at 67 °C for 10 min.
3.7 Immediately place the PCR tubes on ice for at least 5 min.

*Note*: You can also set up a PCR program to all previous three steps while setting the lid temperature to 39 °C as the following.

**Table:** 

37 °C	120 min	Labeling
65 °C	10 min	Heat-inactivation
4 °C	5 min	On ice until the next step

*Note*: At this point you can start the purification of the labeled DNA or store the samples at −20 °C for further purification for later.

### Purification of labeled genomic DNA

4.1 Label the area of a 96-well plate filter-set you are going to use for the purification of the labeled DNA.
4.2 Pre-wet all wells of a 96-well plate filter set with 50.0 μl of 1× TE buffer (pH 8.0).
4.3 Add an extra 35 μl of 1× TE buffer (pH 8.0) to the wells you are going to use for DNA purification.
4.4 Add the whole content of each PCR tube to the wells containing 85 μl of 1× TE buffer (pH 8.0) bringing the total volume of each well to 135 μl.
  Open the valve between the collection flask and the manifold and close the bleed valve (watch the video for visualization).
4.5 Place the plate on the manifold.
4.6 Turn on the house vacuum.
4.7 Press down on the 96-well plate filter set and gently open the valve from the flask to the manifold while checking the pressure indicator that now should start moving toward −20 inches of mercury. Stop opening the valve as soon as the pressure indicator reached to −20 inches of mercury. Using the Silver knob, reduce the pressure and set it between −14 and −16 inches of mercury.
4.8 Set the timer for 10 min.
4.9 After 10 min, close the black valve which connects the vacuum to the manifold and as soon as the pressure indicator reached zero remove the plate. Blot the bottom of the plate by placing it on 5 layers of paper towel.
4.10 Wipe the fluid off the manifold and place the plate on the manifold, and press down on the 96-well plate filter set and gently open the valve from the flask to the manifold while checking the pressure indicator that now should start moving toward −20 inches of mercury. Stop opening the valve as soon as the pressure indicator reached to −20 inches of mercury. Using the silver knob, reduce the pressure and set it between −14 and −16 inches of mercury.
4.11 Set a timer for 10 min.
4.12 After 10 min remove the plate from the vacuum manifold.
4.13 Close all the valves. Open the silver knob and as soon as the pressure indicator reaches zero, gently remove the plate.
4.14 If the wells still appear wet, return the plate on the vacuum for another 5 min.
4.15 Add 35.0 μl 1× TE (pH 8.0) to each well if the labeled DNA will be used for 400 K and 1 million K arrays or add 25.0 μl 1× TE (pH 8.0) to each well if using other types of arrays. Gently place a sheet of plate tape over the sample wells and make sure it is adhered tightly to the top of the wells.

Tap the 96-well plate gently on the bench to make sure that all fluids are at the bottom of the wells and gently place the 96-well plate on the plate vortexer. Cover the plate with aluminum foil and vortex for 10 min at the speed setting of 5.

Gently remove the plate from the vortexer and transfer the content of each well to properly labeled 1.7 ml tubes.

Labeled DNA at this point can be stored for up to 10 days at −20 °C before or after quantification.

### Quantification of the labeled DNA

To measure the amount of labeled DNA, use a UV-vis microvolume spectrophotometer (NanoDrop, NanoDrop Technologies). With its MicroArray setting, the spectrophotometer will measure the Cy3 or Cy5 fluorescence of the labeled genomic DNA and the concentration of total DNA.

5.1 Click on ‘MicroArray’ from the first screen.
5.2 Place 1.6 μl of PCR grade water on the pedestal and click OK.
5.3 From the ‘Sample Type’ menu, choose ‘DNA-50’.
5.4 Using a Kimwipe wipe off the pedestal, gently.
5.5 Place 1.6 μl of 1× TE (pH 8.0) buffer on the pedestal.
5.6 Blank the sample.
5.7 Place 1.6 μl of 1× TE (pH 8.0) buffer on the pedestal and click measure.
5.8 If the reading of fluorescence is between 0.0 and 1.0, you can go ahead and measure your samples, otherwise repeat the steps 5.5–5.7 until the spectrophotometer is properly blanked.
5.9 Measure each sample and print the report.
5.10 Make the best effort to match each test sample with a gender-matched control.

A maximum of 30% variation in the reading for a pair of test and control-labeled DNA is acceptable (for instance if the Cy5 reading of the test is 10.0 and the Cy3 reading of the gender-matched control is 7.0, you can proceed to the next step, otherwise the volume of the test or control sample should be adjusted accordingly).

The purified labeled DNA can be stored for 10 days at −20 °C either before or after quantification.

### Hybridization of patient and control samples to Agilent arrays

For this and all future steps we recommend working in dimmed light and covering your samples with aluminum foil to slow down any possible photobleaching.

*Note*: This step can be done using large-volume strip PCR tubes or large-volume, raised rim 96-well plates along with a thermocycler accepting large volume tubes; alternatively, it can be performed with regular PCR tubes and a heat block which is available in all laboratories. Here we describe use of the heat block and regular 1.5 ml tubes.

6.1 Add the following components as described in Table 2 to a 1.7 ml microcentrifuge tube; Follow the order and scale up for mastermix.

6.2 Mix the sample by pipetting with an SL200 tip. Spin down the tubes at maximum speed for 5 s.
6.3 Lock the tubes with flat Eppendorf tube lock and place them in a heat block at 105 °C and incubate for 3 min.
6.4 Immediately place the tubes in a floating rack, cover with aluminum foil and transfer to a water bath at 37 °C and incubate for 30–40 min.
6.5 Take the sample from the water bath. Spin down for 1 min at maximum speed.

*Note*: When using large-volume string PCR tubes or 96-well plates with a thermocycler, make sure to use string caps. The temperature setting will be 100 °C followed by 37 °C.

### Microarray hybridization

Please note that for most laboratories this will be the rate limiting step as the most common hybridization oven comes with slots for “only” 24 slides. Having a bigger oven or multiple ovens would eliminate this rate limiting step.

Place a SureHyb chamber on the bench and make sure that it is leveled on the working surface.

6.6 Place a clean gasket slide onto the SureHyb chamber. The gasket label (e.g. “Agilent”) must be aligned with the rectangular section of the chamber and must be facing up.
  Load the proper amount of hybridization sample into the pipette tip and avoid generating bubbles. Then gently dispense the hybridization mix into the gasket well. Fill in all wells:
  - For 8 × 60 K arrays, dispense 45 μl of the mixture into each gasket well.
  - For 4 × 180 K arrays, dispense 110 μl of mixture into each gasket well.
  - For 2 × 400 K arrays, dispense 250 μl of mixture into each gasket well.
    Now, you are going to make the sandwich by putting the array slide on top of the gasket slide. The Agilent label on the array slide must face the label of the gasket slide (watch the video for better visualization). With extreme cautions gently place the array slide on top of the gasket slide.
6.7 Gently place the SureHyb chamber cover over the slide sandwich and clamp assembly onto both pieces.
6.8 Hand-tighten the clamp slowly onto the chamber. Tighten enough not to have any leak but not too much to break the glass slide.
6.9 While watching through the sandwich rotate the assembled chamber to see the mobility of bubbles in the chamber. Tap the chamber on the bench gently to free the stuck bubbles.
6.10 Place the assembled chamber in the rotator rack of the hybridization oven in a balanced order and set the temperature to 65 °C. Set the rotator to 10 rpm.
6.11 Hybridize at 65 °C for 40–48 h.
6.12 Proceed to the washing step.

### Washing arrays

In order to speed up the washing section our solution is to prepare several glass jars filled with washing buffers at specified temperatures and to disassemble 4–8 hybridization chamber at a time. Three rounds of washing can be done in 30 min followed by loading all slides into the scanner. Washing more than 8 slides with multiple jars or larger jars did not produce favorable results. However, this step can be sped up if more than one person is involved. (Once again the protocol is designed to be performed by one person throughout). Steps after the assembly of the slide including the washing step are less amenable for automation with current slide designs.

There should be enough space between slides while the mixer magnet is rotating at the bottom of the container. A maximum of 8 slides can be fit in a glass jar with approximate inside dimensions: 70 mm × 185 mm × 90 mm (2.75″ × 4.72″ × 3.54″) and maximum liquid volume of approximately 950 ml (staining dish set (cover, dish & rack), Wheaton^®^ staining dishes, TED Pella Inc, product # 21058). Two people can use two sets of washing units and perform 16 slides at a time for a faster outcome.

7.1 Place 250 ml of Oligo aCGH Wash Buffer 2 in a slide staining jar, add a stir bar, label it as ‘Buffer 2’, cover the jar and then put it in a 37 °C water-bath and incubate for at least 1 h before washing or as soon as it reached 37 °C. (*Note*: You need adequate buffer to cover the slides)
7.2 Pour 350 ml of room-temperature Oligo aCGH Wash Buffer 1 in a slide staining jar; label it as ‘Buffer 1a’, and put it under the hood.
7.3 Pour 300 ml of room-temperature Oligo aCGH Wash Buffer 1 in a slide staining jar; label it as ‘Buffer 1b’, add a stir bar (adding the stir bar is very important to reach excellent quality scores), and put on a magnetic stir plate. (*Note*: You need adequate buffer to cover the slides)
7.4 Fill a slide-staining jar labeled ‘Acetonitrile’ with approximately 250 ml of room-temperature acetonitrile and place it under the hood. (*Note*: You need enough acetonitrile to cover the slides)
7.5 Take the hybridization chambers out of the 65 °C oven for washing.
7.6 Place the hybridization chamber assembly on several layers of paper towel on a flat surface and gently loosen the thumb screw.
7.7 Slide the clamp toward the side and gently take off the cover.
7.8 Using plastic forceps release the array-gasket sandwich from the chamber base and remove it without touching the array surface which is now located on top.
7.9 The slide numeric barcode should face up at this step and you should keep it in the same orientation for the next step.
7.10 By holding the sides of the sandwich with your hand, submerge the sandwich inside the wash buffer 1a jar. (*Note*: Be careful not to touch the array surface)
7.11 Using a plastic forceps and while the sandwich is submerged release the gasket from the sandwich and let it sink.
7.12 Quickly but gently and while holding the slide array from its sides, place the array into a slide rack. (*Note*: Be careful not to touch the array surface)
7.13 Repeat the same step for all other slide arrays and as soon as placing the last slide in the slide rack, move the rack into the wash buffer 1b and set the rotator to 120 rpm. (*Note*: Up to 6 arrays can be placed in a slide rack, but always keep enough distance between two neighboring slides)
7.14 Cover the jar with aluminum foil and set the timer for 5 min.
7.15 30 s before the first washing is done, take the wash buffer 1b jar from the magnetic stir plate to the side under hood.
7.16 Remove the wash buffer 2 jar from the water-bath and place it on the magnetic stir plate, turn off the magnetic rotator.
7.17 Quickly but gently transfer the slide rack from the wash buffer 1b jar to the wash buffer 2 jar located on the magnetic stir plate.
7.18 Set the magnetic rotator to 120 rpm and the timer to 1 min.
7.19 Right after 1 min transfer the slide rack to the acetonitrile jar and set the timer for 1 min.
7.20 With extreme caution and very slowly remove the slide rake from the acetonitrile jar. This step should take about 5–10 s to prevent carrying acetonitrile on the slides.
7.21 Place the rack on a dry and clean surface. (*Note*: Be careful not to touch the array surface)

Scanning the slides immediately after this step is highly recommended to minimize the reduction in the fluorescence signal intensity due to environmental oxidants and possible photobleaching. In case the immediate scanning is not an option keep the slides in a desiccator and in light-sealed boxes (you can use the boxes the array slides come in) or slide holding boxes wrapped in aluminum foil.

*Note*: The used wash buffers cannot be used for more washes; however, acetonitrile can be used for up to 20 slides if the carried wash buffer 2 to the acetonitrile jar is minimal.

Wash buffers can be discarded in the sink and the acetonitrile should be transferred into labeled ‘acetonitrile waste’ container (hood cabinet).

*Note*: To speed up the washing step a high-throughput washing system such as Little Dipper (SciGene, product # 1080-40-1) can be utilized. This system is capable of processing up to 24 slides per run. We were recently informed about this product by our advisers; however, we did not use this platform in the current protocol.

### Preparation of slide array cassette

(These steps are not scalable/high-throughput. These steps require great care and a very steady hand. We recommend practicing these steps with old array slides or microscope slides.)

8.1 Place a clean and dust free cassette on a flat surface and open the lid upward.
8.2 Holding the labeled end of the slide with a plastic forceps or your hand without touching the array surface, place the slide into the cassette.
8.3 Gently close the lid and lock the cassette. (*Note*: Make sure that the array is not touched at any stage).
8.4 Place the cassette into the scanner.

### Scanning slide arrays on the laser scanner

Follow the instruction of the manufacturer (there are many scanners and different software available to choose from). In the following is excitation/emission information for each of the utilized dyes: Cy5, excitation peak is at ∼650 nm, Cy5 emission is at ∼670 nm, and the Cy3 excitation peak is at: ∼550 nm, Cy3 emission peak is at ∼570 nm).

## Results and discussion

**Fig. 1:**
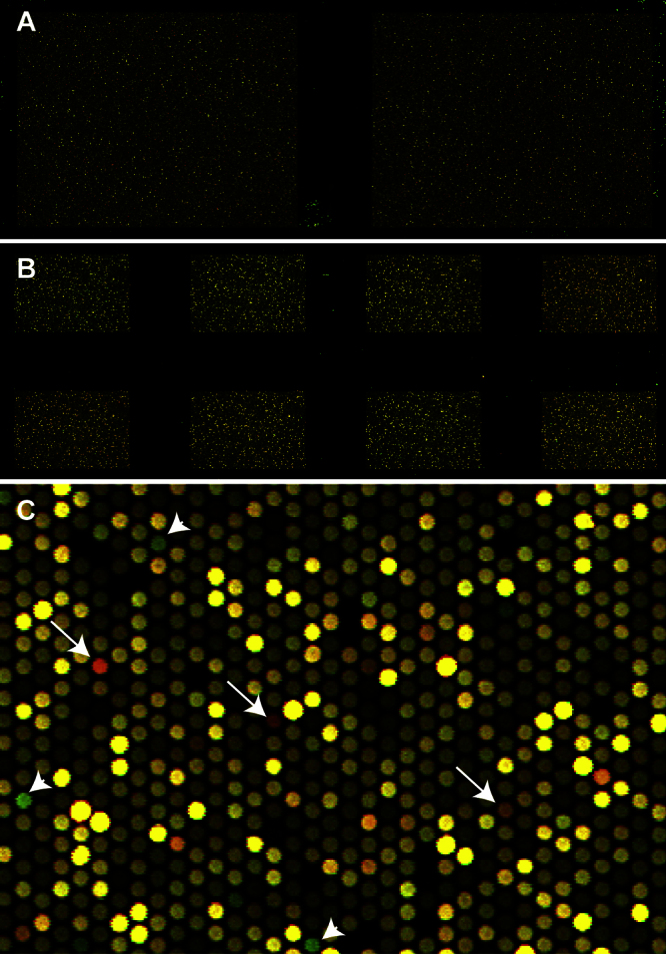
Scanned CNV arrays. A scanned 400 K × 2 (A) and a 60 K × 8 (B) arrays demonstrate thousands of spots represented with different densities of green, red, or yellow color. Higher magnification of a small region of an array (C) demonstrates the variation in color and brightness of each spot. Arrows show the red spots and arrow heads show the green ones. (For interpretation of the references to color in this figure legend, the reader is referred to the web version of this article.)

**Fig. 2:**
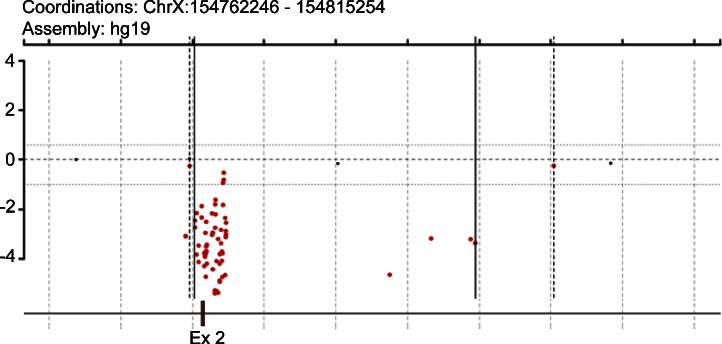
The custom Wed-based software demonstrates a deletion of exon 2 of trimethyllysine hydroxylase, epsilon (TMLHE) located on chromosome X. The *Y*-axis is in logarithmic scale (log 2) and the *X*-axis is the coordinate of the represented segment of DNA (hg 19). Each dot represents the log ratio of the patient/control signal for the specific DNA region.

The scanned array will produce images demonstrating the location and fluorescence density of thousands of spots generated when labeled DNA binds to oligos on the array (Fig. 1). The patient's and the control DNAs are labeled with Cy5 and Cy3 dyes, respectively. The actual color for Cy5 and Cy3 emissions are in the red and green/yellow spectrum, respectively. In our array we assign red for Cy5-labeled DNA and green for Cy3-labeled DNA. Accordingly, when Cy5- and Cy3-labeled DNA are balanced the spot appears yellow. Excess of Cy5-labled DNA (patient) for any specific target appears red suggesting a duplication event in that region. Similarly, reduced Cy5-labled DNA (patient) for any specific target appears green suggesting a deletion event in that region (Fig. 1). Various image processing software can then be used to process the image data and provide copy number variations in patient's DNA. The final analyzed data is demonstrated as the Cy5/Cy3 ratio (log ratio) for each target. A positive log ratio indicates excess of Cy5-labled DNA which happens in a duplication event and a negative log ratio indicates reduction of Cy5-labeled DNA which happens in a deletion event. In our graphs the positive log ratios are demonstrated as green and negative log ratios as red dots. We used both Agilent software package and a custom Web-based tool [12] in our experiments (Fig. 2). A representative plot demonstrates a deletion of exon 2 of trimethyllysine hydroxylase, epsilon (TMLHE) gene located on chromosome X (Fig. 2) [14], [15].

The focus of this paper was to introduce a high-throughput solution for performing aCGH/CMA with high resolution and quality. We managed to reach high quality arrays with 0.16 ± 0.06 (mean ± SD, *n* = 48) Derivative Log Ratio (DLR) spread, a measure of array quality (<0.20 considered as Excellent) for our custom array designs and a 17 ± 0.04 (mean ± SD, *n* = 90) DLR for version 9 BCM clinical arrays. Values less than 0.2 DLR are considered excellent and in our hand we performed repeated excellent quality demonstrated with narrow standard deviation of the DLR. A representative quality control report with average DLR can be seen in the supplementary material.

## Figures and Tables

**Table 1:** The labeling master mix for single tube reaction labeling. Scale up for more reactions. If you used heat-fragmentation use the suggested volumes as presented in the table. If you used the enzyme digestion protocol all the volumes should be divided by 2.

Labeling master mix		
		Volume (μl)
1	dNTP (1.2 mM of dATP, dGTP, dTTP & 0.6 mM of dCTP	5.0
2	Cy3 or Cy5 labeled dye (labeled dCTP of 1.0 mM)	3.0
3	Klenow fragment (40 units/μl)	1.0

	Total volume	9.0

**Table 2:** The hybridization master mix for single tube reaction labeling. All volumes in “μl”.

	Component	8 × 60 K	4 × 180 K	2 × 400 K
1	Cy5-labeled DNA	20	20	35
2	Cy3-labeled DNA	20	20	35
3	1X TE (pH 8.0)	0	0	10
4	CoT-1 DNA (1.0 mg/ml)	3	5	25
5	Agilent 10× Blocking agent	5	11	26
6	Agilent 2× Hybridization buffer	22	55	130

	Total volume	70	110	260
