# Supplementary figures and images for: Chromosomal microarray analysis, or comparative genomic hybridization: A high throughput approach
Source: MethodsX. 2015 Dec 2;3:8–18. doi: 10.1016/j.mex.2015.11.005 (PMC4707176; doi:10.1016/j.mex.2015.11.005)

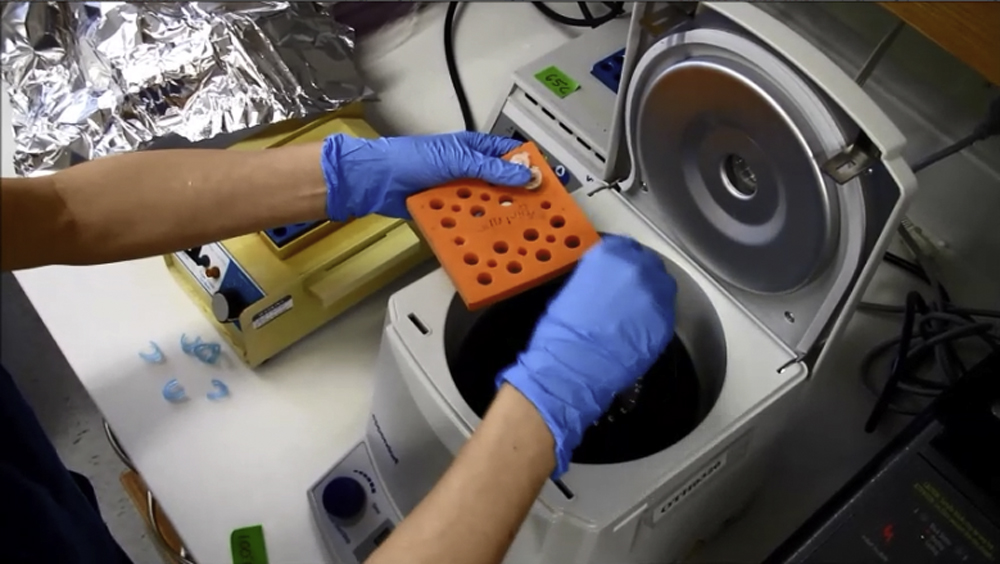

Supplement: Supplementary file 1 [file mmc1.jpg]
