# Supplementary material for: Chromosomal microarray analysis, or comparative genomic hybridization: A high throughput approach
Source: MethodsX. 2015 Dec 2;3:8–18. doi: 10.1016/j.mex.2015.11.005 (PMC4707176; doi:10.1016/j.mex.2015.11.005)
Supplement: Supplementary file 2 [file mmc2.pdf]

QC Report - Agilent Technologies : 2 Color CGH

Date	Friday, January 17, 2014 - 15:26	Sample(red/green)	
User Name	Administrator	FE Version	11.5.1.1
Image	US45103095_256261710002_S01 [2_2]	BG Method	Detrend on (NegC)
Protocol	CGH_1105_Oct12 (Read Only)	Multiplicative Detrend	True
Grid	SMH_AU_Conf_110_062617_D_F_20131203	Dye Norm	Linear
Saturation Value	65508 (r), 65507 (g)		
DyeNorm List	NA		
No of Probes in DyeNorm List	NA		

Spot Finding of the Four Corners of the Array

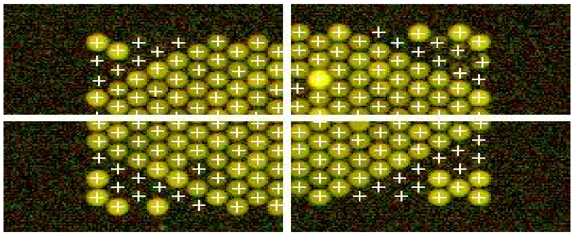

Grid Normal

Outlier Numbers with Spatial Distribution

384 rows x 164 columns

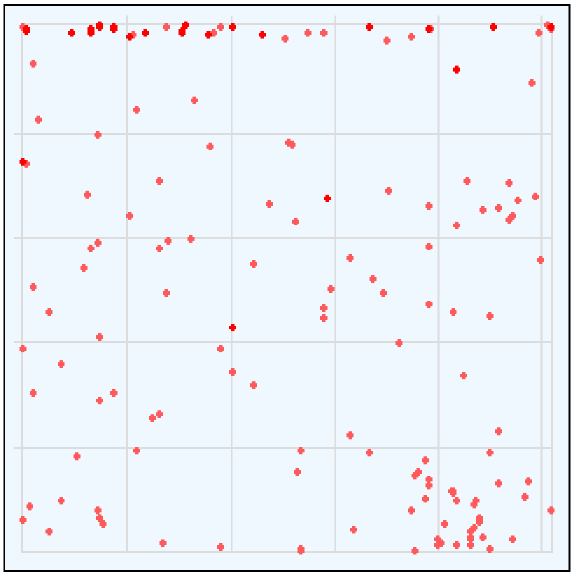

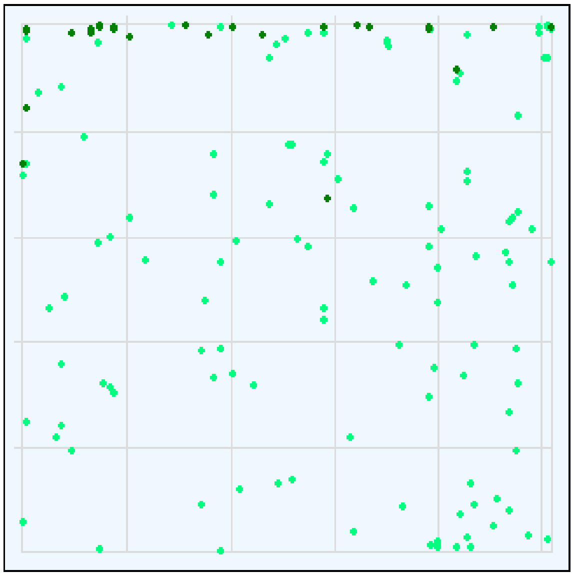

•Red FeaturePopulation •Red Feature NonUniform
•Green FeaturePopulation •Green Feature NonUniform

Evaluation Metrics for CGH_QCMT_Oct12
Excellent (8) ; Good (3) ; Evaluate (1)

Metric Name	Value	Excellent	Good	Evaluate
IsGoodGrid	1.00	>1	NA	<1
AnyColorPrcntFeatNonU...	0.05	<1	1 to 5	>5
DerivativeLR_Spread	0.16	<0.20	0.20 to 0.30	>0.30
gRepro	0.05	0 to 0.05	0.05 to 0.20	<0 or >0.20
g_BGNoise	5.29	<5	5 to 15	>15
g_Signal2Noise	121.92	>100	30 to 100	<30
g_SignalIntensity	645.33	>150	50 to 150	<50
rRepro	0.05	0 to 0.05	0.05 to 0.20	<0 or >0.20
r_BGNoise	8.33	<5	5 to 15	>15
r_Signal2Noise	100.19	>100	30 to 100	<30
r_SignalIntensity	834.33	>150	50 to 150	<50
RestrictionControl	-1.00		0.80 to 1	<0.80 or >1

• Excellent • Good • Evaluate

Histogram of Signals Plot (Red)

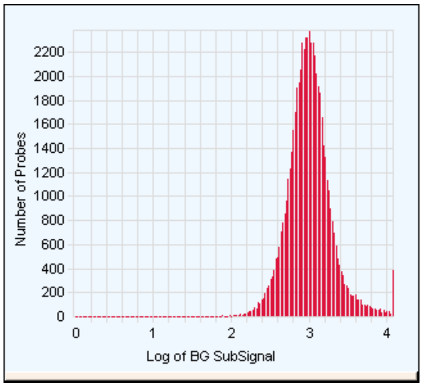

Histogram of Signals Plot (Green)

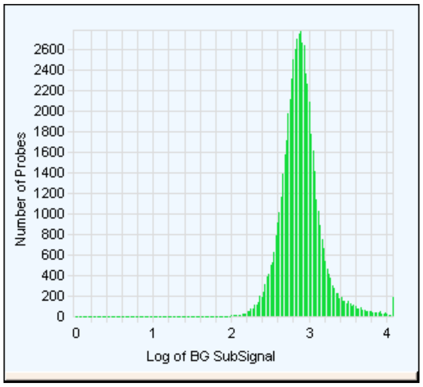

Note: The restriction control is flagged for evaluation. This is due to the fact that the heat fragmentation was used instead of enzyme restriction.

Feature	Red	Green	Any	%Outlier
Non Uniform	25	25	29	0.05
Population	132	122	211	0.34

Spatial Distribution of the Positive and Negative LogRatios

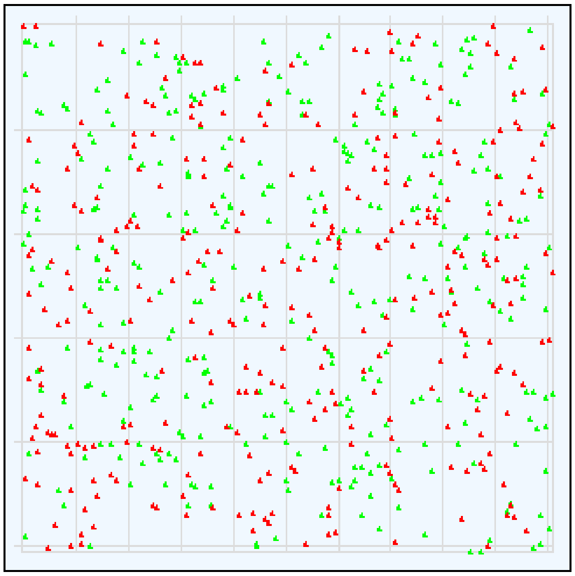

#Positive:308 (Red) ; #Negative:348 (Green)

● Positive ● Negative

Positive: 0.52% of NonCtrl Features : Random (Value 0.96)
Negative: 0.59% of NonCtrl Features : Random (Value 1.13)

Red and Green Background Corrected Signals (Non-Control Inliers)

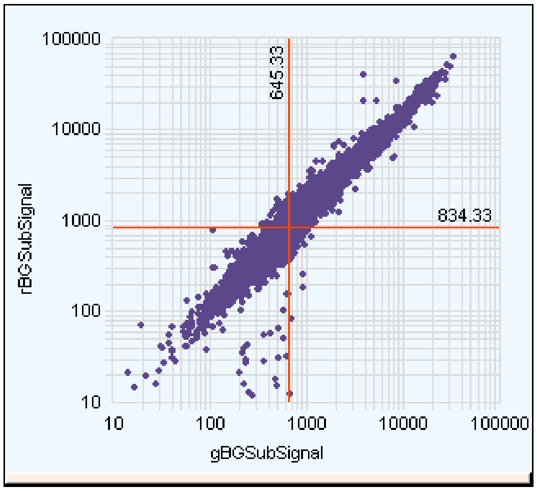

Features (NonCtrl) with BGSubSignals < 0: 1 (Red); 0 (Green)
